# Relationship between coagulation function indicators and neurological outcome following rt-PA thrombolysis in acute ischaemic stroke

**DOI:** 10.3389/fmed.2026.1965269

**Published:** 2026-10-21

**Authors:** Jing Han, Quping Ouyang, Yue Li

**Affiliations:** Department of Neurology, Beijing Shunyi Hospital, Beijing, China

**Keywords:** activated partial thromboplastin time, acute ischemic stroke, D-dimer, fibrinogen, neurological outcome, prothrombin time, rt-PA thrombolysis

## Abstract

**Aim:**

To assess the relationship between baseline coagulation parameters and 90-day neurological functional status in patients with acute ischaemic stroke (AIS) undergone intravenous recombinant tissue plasminogen activator (rt-PA) thrombolysis.

**Methods:**

We implemented a retrospective cohort study of 200 AIS patients who underwent intravenous rt-PA thrombolysis from January 2024 to December 2025. Baseline coagulation parameters were measured before thrombolysis. The primary outcome was poor functional outcome [modified Rankin Scale (mRS) score 3–6 at 90 days]. Multivariable logistic regression was used to estimate adjusted associations between baseline variables and 90-day outcome. A four-marker logistic model was explored, and receiver operating characteristic (ROC) analysis was used to describe its apparent discrimination within the study cohort.

**Results:**

Of the 200 enrolled patients, poor 90-day functional status was seen in 68 (34.0%). The baseline D-dimer (2.18 ± 1.64 vs. 0.87 ± 0.65 mg/L, *P* < 0.001), fibrinogen (3.82 ± 0.91 vs. 3.15 ± 0.76 g/L, *P* < 0.001), PT (12.84 ± 1.12 vs. 12.15 ± 0.89 s, *P* < 0.001) and APTT (32.56 ± 3.42 vs. 29.87 ± 2.65 s, *P* < 0.001) levels were higher in the poor outcome group than the favourable outcome group. In multivariable analysis, elevated D-dimer (OR = 2.143, 95% CI = 1.452–3.162, *P* < 0.001), increased fibrinogen (OR = 1.876, 95% CI = 1.123–3.134, *P* = 0.016) and prolonged APTT (OR = 1.215, 95% CI = 1.032–1.431, *P* = 0.019) remained associated with poor 90-day functional status after adjustment for the measured covariates included in the model. The four-coagulation-marker model had an apparent AUC of 0.812 (95% CI = 0.735–0.888) in the same cohort.

**Conclusion:**

Baseline D-dimer, fibrinogen and APTT were associated with 90-day neurological functional status among AIS patients treated with intravenous rt-PA. These markers should not be interpreted as stand-alone predictors of outcome; their incremental prognostic value beyond established clinical factors and their clinical utility require prospective external validation.

## Introduction

Acute ischaemic stroke (AIS) is a common clinical cerebrovascular disease, and pathologically is characterised by focal ischaemic induced brain tissue necrosis (1). It is featured by acute onset, a variety of complications, high rates of disability and mortality along with poor prognosis (2). At present, the main therapy for acute phase AIS is intravenous thrombolysis with recombinant tissue plasminogen activator (rt-PA), which can achieve a good therapeutic effect (3). Nevertheless, despite timely thrombolytic therapy, unfavourable long-term functional status is seen in about one-third of patients. The underlying mechanisms are complex, involving thrombus load, reperfusion injury, imbalance of coagulation-fibrinolysis and inflammatory response (4).

The coagulation-fibrinolysis system is of crucial importance for the pathophysiology of AIS and thrombolytic therapy (5). The formation and dissolution of thrombus are complex interactions involving coagulation factors, fibrinolytic proteins and endothelial function (6). The conventional coagulation parameters, such as D-dimer, fibrinogen, prothrombin time (PT) along with activated partial thromboplastin time (APTT), which are widely available and routinely tested, reflect different aspects of the coagulation cascade (7).

Elevated D-dimer levels indicate increased fibrinolytic activity and thrombus burden (8). Previous studies have demonstrated that increased D-dimer at admission and after thrombolysis is linked to an elevated risk of hemorrhagic transformation and poor functional status in AIS patients undergone rt-PA (9). Fibrinogen is the precursor of fibrin, which is involved in blood viscosity, inflammation, and thrombus formation. Changes in fibrinogen levels during thrombolysis have been linked to hemorrhagic complications and prognosis of AIS patients (10).

PT and APTT are used to detect the exogenous and endogenous pathways of blood coagulation respectively. Although these are basic coagulation screening tests, their prognostic value in the setting of rt-PA thrombolysis has not been fully explored. Most previous studies focused on single markers or dynamic changes after thrombolysis, and comprehensive evaluation of baseline conventional coagulation panels is lacking.

This retrospective cohort study was designed to examine whether routinely available coagulation parameters measured before treatment were associated with 90-day neurological status specifically among AIS patients receiving intravenous rt-PA.

## Methods

### Study design and participants

A retrospective observational cohort study was implemented within our hospital. Consecutive eligible AIS patients undergone intravenous rt-PA (Actilyes, Boehringer Ingelheim of Germany, Total dose: 0.9 mg/Kg. Maximum dose: 90 mg. 10% of the total dose is administered intravenously in 1 min, and the remaining 90% is infused by a micro-pump within 1 h.) thrombolysis from January 2024 to December 2025 were screened. After 24 h of thrombolysis, if the head CT examination confirmed no cerebral hemorrhage, the patient was orally administered 100 mg of aspirin + 75 mg of clopidogrel hydrochloride per day for a total of 21 days, and was also treated with brain protectants, lipid-lowering drugs, and drugs to improve circulation. Finally, 200 patients were eligible and included in the analysis. The study was approved by the hospital ethics committee, and informed consent was waived owing to the retrospective nature of the study. Baseline demographic and clinical covariates were extracted from the electronic medical record, including age, sex, baseline NIHSS score, onset-to-needle time, hypertension, diabetes mellitus, dyslipidaemia, atrial fibrillation, current smoking status, and TOAST subtype.

Inclusion criteria: (1) Clinical diagnosis of AIS according to CT or MRI of brain; (2) All patients had the first ever attack of the disease; (3) The patient had definite indications for intravenous thrombolysis treatment; (4) Complete follow-up data for 90 days.

Exclusion criteria: (1) Patients with a known history of intracranial hemorrhage or suspected intracranial hemorrhage; (2) Presence of coagulation disorders or immune diseases; (3) Receipt of effective oral anticoagulant therapy; (4) Dysfunction of vital organs including the liver and kidneys; (5) Severe psychiatric or cognitive impairment. For clarity, the intracranial haemorrhage exclusion criterion referred to known or suspected intracranial haemorrhage before rt-PA administration; patients were not excluded solely because haemorrhagic transformation occurred after thrombolysis.

### Coagulation function tests

Immediately on arrival at the hospital, venous blood was drawn into tubes containing sodium citrate as an anticoagulant prior to administration of rt-PA. The plasma was separated by centrifugation, and the coagulation parameters (D-dimer, fibrinogen, PT, APTT) were determined via an automated coagulation analyser (Sysmex CS-5100, Sysmex Corporation, Kobe, Japan).

### Outcome measures

The primary outcome was poor 90-day neurologic functional status, defined as a modified Rankin Scale (mRS) score of 3–6 at 90 days after symptom onset. The mRS assessment was performed by two board-certified neurologists trained on the mRS grading criteria in a standardised way. Follow-up was by face-to-face outpatient interview or structured telephone interview. To minimise observer bias, all assessors were blinded to the baseline coagulation test results of the included patients. Head CT was performed at approximately 24 h after thrombolysis as part of routine post-thrombolysis management. Hemorrhagic transformation was not used to exclude patients on the basis of subsequent functional outcome; however, hemorrhagic transformation subtypes and symptomatic intracranial haemorrhage were not systematically adjudicated as separate study endpoints in this retrospective analysis.

Secondary outcomes were: Comparison of baseline coagulation parameters between groups; Independent predictors of poor 90-day functional outcome; Predictive performance of single and combined coagulation parameters for poor functional outcome.

### Statistical analysis

Statistical analysis was performed via SPSS version 26.0. The Shapiro–Wilk test was employed for assessing the normality of continuous variables. Normally distributed data were expressed as mean ± standard deviation (*x¯* ± *s*) and compared by independent-samples *t* test. Non-parametric data were presented as median (interquartile range) and compared with the Mann–Whitney *U* test. Categorical variables were expressed as frequencies (percentages) and analysed via the chi-square test or Fisher's exact test. Variables with *P* < 0.10 in univariate analyses were considered for multivariable analysis together with clinically relevant covariates. The final multivariable model reported in Table 2 included age, baseline NIHSS, atrial fibrillation, D-dimer, fibrinogen, PT and APTT. In the exploratory four-marker model, D-dimer, fibrinogen, PT and APTT were entered as continuous independent variables and poor 90-day functional status as the dependent variable. The predicted probability from this model was used to construct the ROC curve. All statistical tests were two-sided and *P* < 0.05 was considered statistically significant. Baseline NIHSS was analysed as a continuous covariate because stroke severity is a major determinant of 90-day functional outcome. No *post hoc* NIHSS restriction (for example, 7–20 points) was applied, because all included patients had a definite indication for intravenous thrombolysis and arbitrary truncation of the NIHSS range would reduce sample size and may introduce selection bias.

## Results

### Baseline characteristics

The poor outcome group was older (*P* < 0.001), had higher baseline NIHSS scores (*P* < 0.001), and had higher proportions of atrial fibrillation (*P* = 0.001) and hypertension (*P* = 0.007) than the favourable outcome group. The two groups were comparable in the aspects of sex, diabetes mellitus, dyslipidaemia, smoking history, onset-to-needle time, and TOAST subtype distribution (all *P* > 0.05) (Table 1).

**Table 1:** Baseline clinical characteristics of patients with favorable and poor 90-day outcomes.

Variable	Favorable outcome (*n* = 132)	Poor outcome (*n* = 68)	*t*/*χ*^2^/*Z*	*P* value
Age (years, *x¯* ± *s*)	63.8 ± 11.4	69.7 ± 10.5	3.559	<0.001
Male sex, *n* (%)	85 (64.4)	41 (60.3)	0.323	0.569
Baseline NIHSS, median (IQR)	6 (4–9)	12 (8–16)	7.835	<0.001
Onset-to-needle time (min, *x¯* ± *s*)	152.6 ± 48.3	158.4 ± 51.2	0.788	0.431
Hypertension, *n* (%)	78 (59.1)	53 (77.9)	7.057	0.007
Diabetes mellitus, *n* (%)	35 (26.5)	22 (32.4)	0.750	0.386
Dyslipidemia, *n* (%)	42 (31.8)	25 (36.8)	0.492	0.482
Atrial fibrillation, *n* (%)	25 (18.9)	27 (39.7)	10.087	0.001
Current smoking, *n* (%)	41 (31.1)	18 (26.5)	0.472	0.492
TOAST classification, *n* (%)			2.154	0.541
LAA	68 (51.5)	32 (47.1)		
CE	31 (23.5)	21 (30.9)		
SVO	26 (19.7)	11 (16.2)		
Other/undetermined	7 (5.3)	4 (5.9)		

### Baseline coagulation parameters

Patients with poor 90-day outcomes had significantly higher D-dimer level, fibrinogen level, prolonged PT and APTT relative to those with favourable outcomes (all *P* < 0.001, Figure 1).

**Figure 1:**
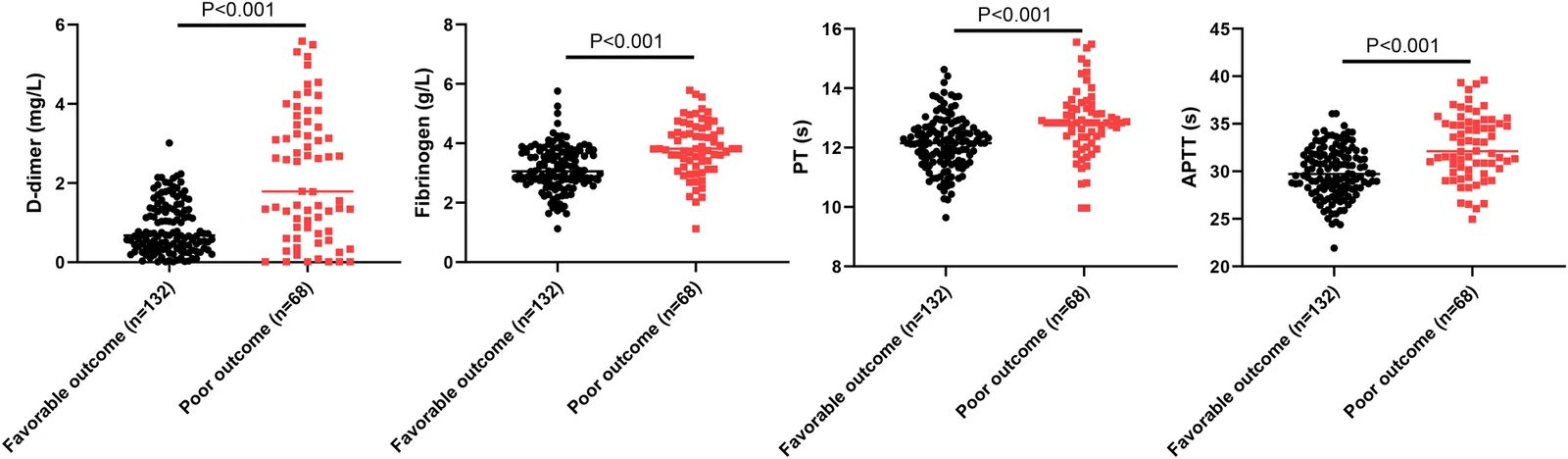
Comparison of baseline coagulation parameters between the two groups.

### Multivariate logistic regression analysis

As shown in Figure 2 and Table 2, higher baseline D-dimer (OR = 2.143, 95% CI = 1.452–3.162, *P* < 0.001), elevated fibrinogen (OR = 1.876, 95% CI = 1.123–3.134, *P* = 0.016) and longer APTT (OR = 1.215, 95% CI = 1.032–1.431, *P* = 0.019) remained associated with poor 90-day functional status after adjustment for the covariates included in the model. PT was not statistically significant (OR = 1.187, 95% CI = 0.896–1.573, *P* = 0.232). Older age and higher baseline NIHSS score were also independently associated with poor outcome (OR = 1.043, 95% CI = 1.005–1.082, *P* = 0.027 and OR = 1.240, 95% CI = 1.131–1.359, *P* < 0.001, respectively).

**Figure 2:**
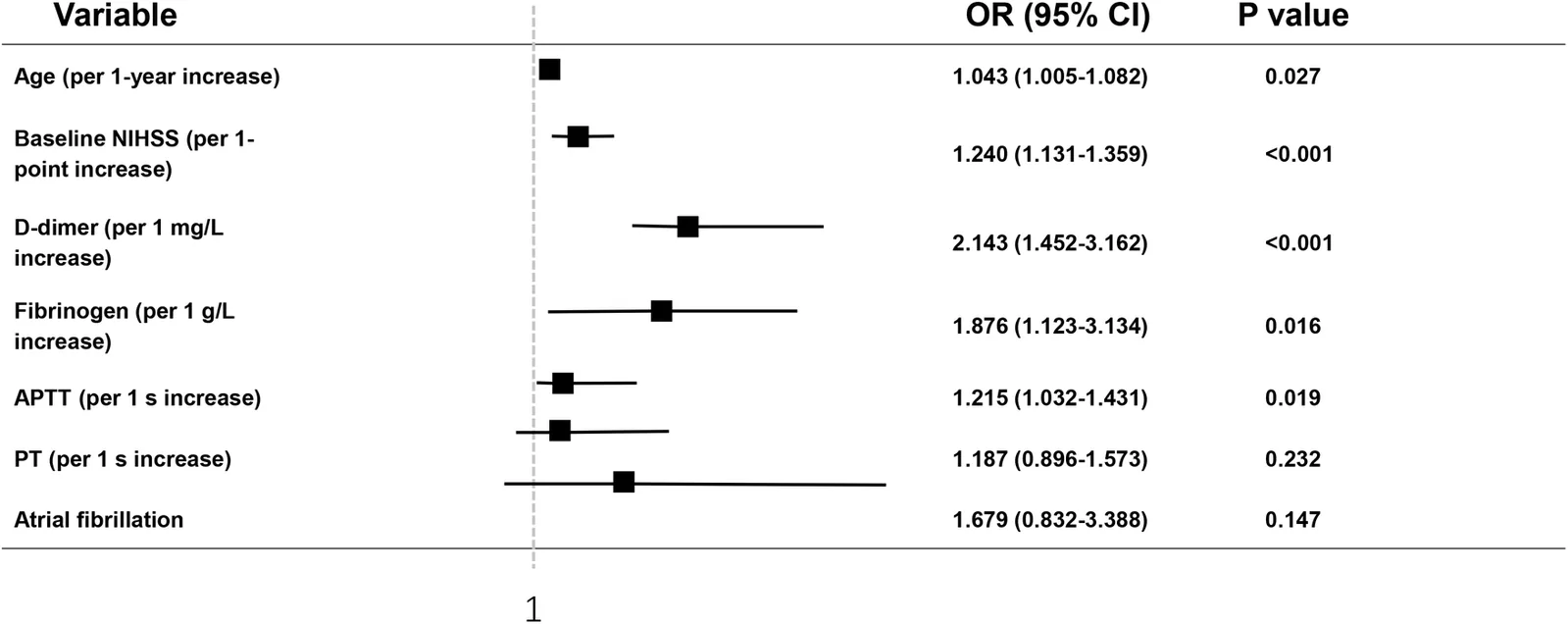
Forest plot of multivariate logistic regression analysis for poor 90-day functional outcome.

**Table 2:** Multivariate logistic regression analysis of factors associated with poor 90-day functional outcome.

Variable	*β*	SE	Wald *χ*^2^	OR (95% CI)	*P* value
Age (per 1-year increase)	0.042	0.019	4.892	1.043 (1.005–1.082)	0.027
Baseline NIHSS (per 1-point increase)	0.215	0.047	20.936	1.240 (1.131–1.359)	<0.001
D-dimer (per 1 mg/L increase)	0.762	0.199	14.658	2.143 (1.452–3.162)	<0.001
Fibrinogen (per 1 g/L increase)	0.629	0.263	5.718	1.876 (1.123–3.134)	0.016
APTT (per 1 s increase)	0.195	0.084	5.407	1.215 (1.032–1.431)	0.019
PT (per 1 s increase)	0.171	0.144	1.413	1.187 (0.896–1.573)	0.232
Atrial fibrillation	0.518	0.357	2.104	1.679 (0.832–3.388)	0.147

### ROC curve analysis

All four individual coagulation parameters showed modest apparent discrimination for poor 90-day functional status, with AUC values of 0.703–0.728 (Figure 3 and Table 3). Among the individual markers, APTT had an AUC of 0.728 (95% CI = 0.651–0.804), D-dimer 0.726 (95% CI = 0.640–0.811), fibrinogen 0.723 (95% CI = 0.646–0.800), and PT 0.703 (95% CI = 0.624–0.782). The exploratory four-marker model had an AUC of 0.812 (95% CI = 0.735–0.888), higher than the AUCs of the individual markers (all *P* < 0.05). Because these estimates were obtained in the same cohort used to construct the model, they should be regarded as apparent discrimination rather than validated clinical predictive performance.

**Figure 3:**
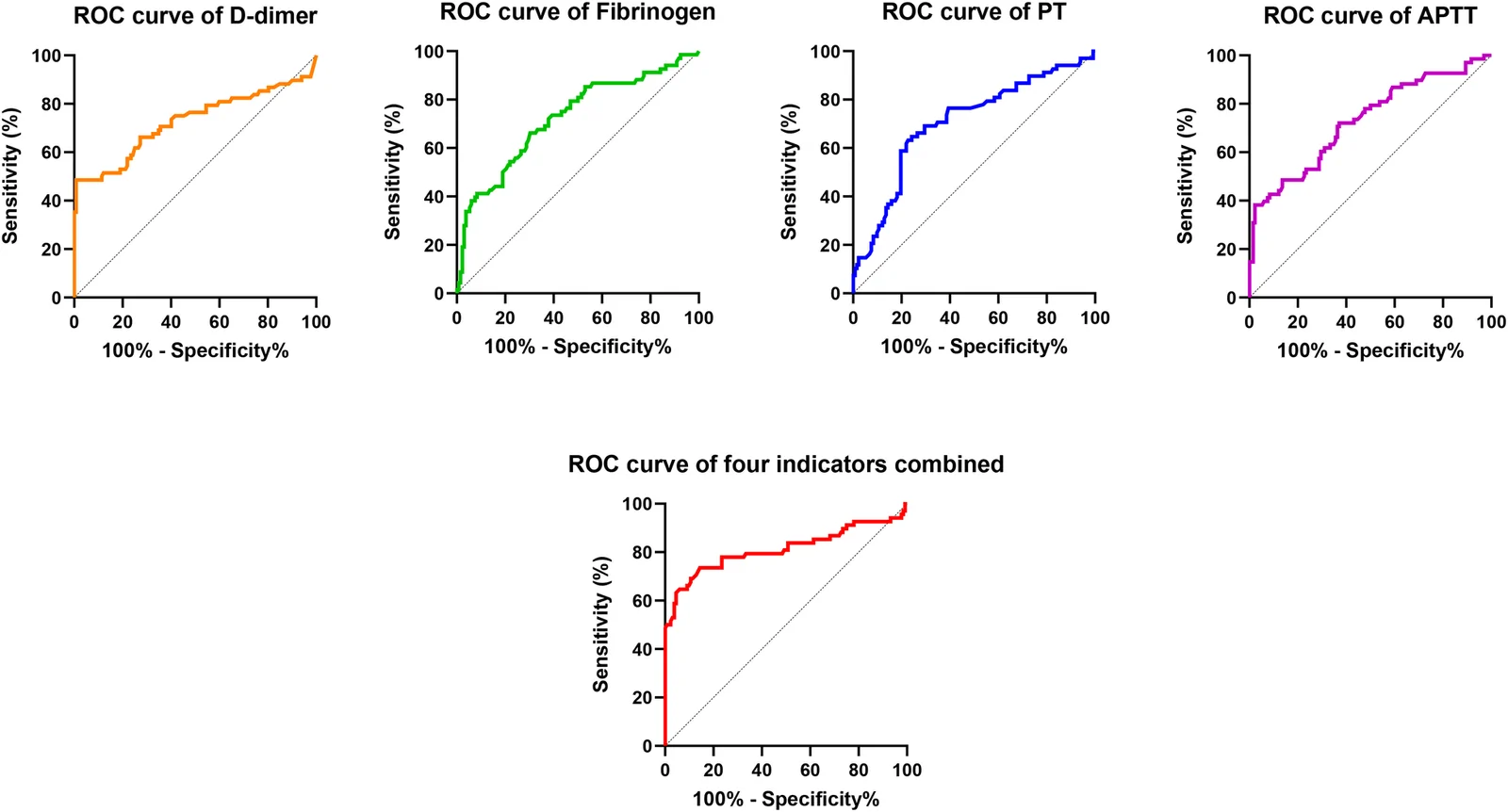
ROC curve analysis of coagulation indicators for predicting poor 90-day functional outcome.

**Table 3:** ROC curve analysis of coagulation indicators for predicting poor 90-day functional outcome.

Indicator	AUC	95% CI	Optimal cutoff	Sensitivity (%)	Specificity (%)	Youden index
D-dimer	0.726	0.640–0.811	1.25 mg/L	70.6	75.8	0.464
Fibrinogen	0.723	0.646–0.800	3.52 g/L	64.7	72.0	0.367
PT	0.703	0.624–0.782	12.55 s	61.8	69.7	0.315
APTT	0.728	0.651–0.804	31.20 s	67.6	71.2	0.388
Four indicators combined	0.812	0.735–0.888	–	76.5	78.8	0.553

To further assess whether coagulation parameters provided additional prognostic information beyond established clinical factors, we compared a clinical model, a coagulation model, and a combined model (Table 4 and Figure 4). The clinical model incorporating age, baseline NIHSS, hypertension, and atrial fibrillation yielded an AUC of 0.853 (95% CI, 0.798–0.908). The coagulation model comprising D-dimer, fibrinogen, PT, and APTT yielded an AUC of 0.812 (95% CI, 0.751–0.873). The combined model incorporating both clinical and coagulation variables showed the highest discrimination, with an AUC of 0.892 (95% CI, 0.845–0.939). DeLong testing showed no significant difference between the clinical and coagulation models (*P* = 0.254), whereas the combined model demonstrated greater discrimination than both the clinical model (*P* = 0.007) and the coagulation model (*P* = 0.0008). These findings suggest that coagulation parameters may provide incremental prognostic information when considered together with established clinical factors.

**Table 4:** Comparison of clinical, coagulation, and combined prognostic models.

Model	Variables	AUC (95% CI)	Sensitivity/specificity	DeLong comparison
Clinical	Age + NIHSS + hypertension + AF	0.853 (0.798–0.908)	79.4%/77.3%	vs. Combined: *P* = 0.007
Coagulation	D-dimer + fibrinogen + PT + APTT	0.812 (0.751–0.873)	82.4%/66.7%	vs. Combined: *P* = 0.0008
Combined	Clinical + coagulation variables	0.892 (0.845–0.939)	80.9%/88.6%	Reference

Additional DeLong comparison: clinical vs. coagulation, *P* = 0.254.

**Figure 4:**
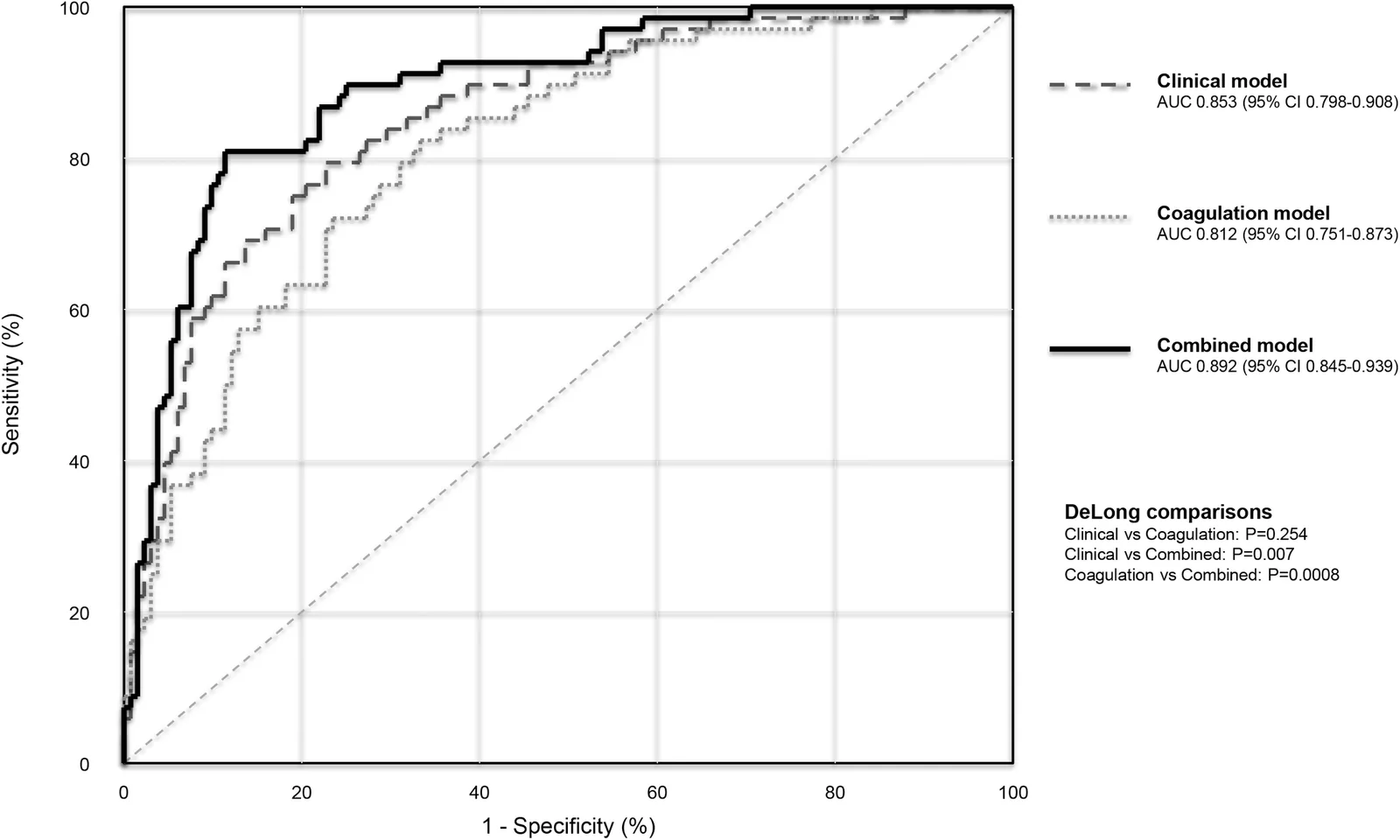
ROC curve comparison of the clinical, coagulation, and combined prognostic models.

## Discussion

The results show that D-dimer, fibrinogen and APTT remained associated with poor 90-day functional status after adjustment for the measured covariates included in Table 2, including age, baseline NIHSS and atrial fibrillation. These adjusted associations are the principal finding of the study, but residual confounding remains possible in this retrospective single-centre cohort. AIS itself represents an acute thrombo-inflammatory and physiological stress state; therefore, admission coagulation abnormalities may reflect both antecedent haemostatic status and stroke-related activation of coagulation and fibrinolysis. Because historical coagulation reports before the index stroke were not consistently available in this retrospective dataset, the present study cannot determine whether an abnormal admission value was chronic or newly induced by the acute event. Accordingly, the findings should be interpreted as associations between pre-thrombolysis coagulation values and 90-day functional outcome rather than evidence of pre-existing chronic coagulopathy.

Of all coagulation indicators, baseline D-dimer was most strongly and independently linked to poor 90-day status in our study (OR = 2.143, *P* < 0.001). This result is in accordance with previous work by Jin and colleague who showed that an increased D-dimer during follow-up after thrombolysis was an independent indicator of unfavourable functional status, with an AUC of 0.760 (11). Several mechanisms may be involved in the association of elevated D-dimer with worse status. Higher baseline D-dimer levels reflect greater thrombus burden and more extensive thrombotic activation, which may lower the likelihood of successful recanalisation and increase the risk of re-thrombosis (12). Second, high D-dimer is often linked to cardioembolic stroke aetiology which usually has more severe neurological deficits and higher complication rates (13). Finally, D-dimer also reflects systemic inflammatory response and endothelial dysfunction contributing to secondary brain injury after ischemia-reperfusion (14).

We found that high baseline fibrinogen was independently linked to poor 90-day status (OR = 1.876, *P* = 0.016). This observation is consistent with a recent meta-analysis which suggested that elevated fibrinogen level at baseline might be an independent indicator of short-term poor functional status in elderly AIS patients (15). Similarly, Mehta et al. also proposed that fibrinogen (OR = 1.011, *P* < 0.001) was an independent indicator of early neurological deterioration in AIS patients (16). This may be due to the high fibrinogen level results in increased blood viscosity and erythrocyte aggregation, with adverse effects on microcirculatory perfusion in the ischaemic penumbra (17). In addition, fibrinogen participates in inflammatory processes by enhancing the adhesion of leukocytes and platelet aggregation, which aggravate neuroinflammation and blood-brain barrier breakdown (18).

Univariate analysis showed that PT and APTT, representing the extrinsic and intrinsic pathways of coagulation, respectively, were prolonged in the poor outcome group. Nevertheless, only APTT was an independent predictor, and PT was no longer statistically significant in multivariate analysis. Thus, activation of the intrinsic coagulation pathway may be more relevant to stroke severity and prognosis during rt-PA thrombolysis. A prolonged APTT may be a manifestation of pre-existing activation of coagulation, endothelial dysfunction or underlying systemic conditions that negatively impact treatment response and recovery. The lower prognostic value of PT may be due to its lower sensitivity to minor coagulation abnormalities and the greater dependence on liver synthetic function.

Dynamic haemostatic changes after thrombolysis are also clinically relevant. Previous studies have shown that post-thrombolysis D-dimer elevation and fibrinogen depletion are associated with symptomatic intracranial haemorrhage and poor functional outcome (10, 11). These data support serial post-treatment testing when the research question concerns thrombolysis-related coagulopathy or haemorrhagic transformation. Our study addressed a different, narrower question: whether routinely available coagulation values obtained before rt-PA were associated with later functional status. We have therefore avoided combining non-standardised post-treatment measurements with the baseline model and now state this distinction explicitly.

The relevance of the thrombolysis setting in this study is primarily clinical and population-specific rather than causal. All coagulation measurements were obtained before rt-PA, so the present analysis does not test whether rt-PA caused the coagulation abnormalities or whether the marker-outcome associations are unique to thrombolysed patients. Instead, it asks whether information already available before treatment is associated with later outcome among patients who actually receive intravenous thrombolysis. This is clinically relevant because such baseline information is available at the point of acute treatment, while the subsequent haemostatic environment is modified by rt-PA. A comparison with non-thrombolysed stroke patients could address whether the associations are treatment-specific, but that would constitute a different comparative-effect question and would require careful control of confounding by indication, because patients who do and do not receive thrombolysis differ systematically in eligibility, time window, contraindications and stroke characteristics. The present four-marker model should therefore be viewed as exploratory. Although its apparent AUC was 0.812, it was developed and evaluated in the same cohort and was not compared with a clinical-only model containing established prognostic factors. Consequently, this study does not establish incremental prognostic value or clinical utility of the coagulation panel beyond age, NIHSS and other clinical information.

The present study has several limitations. First, this was a single-centre retrospective study with a relatively small sample size; the cohort consisted of consecutive eligible patients during the prespecified study period, and the findings require confirmation in larger multicentre cohorts. Baseline differences in age, NIHSS, hypertension and atrial fibrillation also indicate important case-mix differences between outcome groups. Although multivariable adjustment was performed for the covariates reported in Table 2, residual confounding, including confounding by measured factors not retained in the final model and by unmeasured factors, cannot be excluded. Second, the study included only patients who received intravenous rt-PA and had no non-thrombolysed comparison group. Therefore, the findings apply to this treated population and cannot determine whether the observed associations are specific to thrombolysis or are general prognostic associations in AIS. Future comparative cohorts could examine thrombolysed and non-thrombolysed patients using rigorous methods to address confounding by indication. Third, only pre-thrombolysis coagulation parameters were analysed. Historical coagulation reports before the index stroke and standardised serial measurements at approximately 48 h after rt-PA were not consistently available, so we could not distinguish chronic coagulation abnormalities from acute stroke-related changes or characterise post-thrombolysis dynamics. Fourth, a complete set of biochemical indicators was not incorporated into the prognostic model. We attempted to reduce major systemic confounding by excluding patients with known coagulation disorders, effective oral anticoagulant therapy, immune diseases, and vital-organ dysfunction including liver and kidney dysfunction; nevertheless, residual systemic confounding remains possible. Fifth, although patients were not excluded because of post-thrombolysis haemorrhagic transformation, haemorrhagic transformation subtype and symptomatic intracranial haemorrhage were not systematically adjudicated as separate endpoints, preventing assessment of whether bleeding complications mediated the association between coagulation markers and 90-day outcome. Sixth, information on recanalisation status and infarct volume was unavailable. Finally, the four-marker model was developed and evaluated in the same dataset without external validation, calibration assessment, or formal evaluation of incremental value over a clinical-only model. Its AUC should therefore be considered an apparent measure of discrimination rather than evidence of established clinical utility. Future multicentre prospective studies should include serial coagulation testing, biochemical markers, formal haemorrhagic-transformation adjudication, comparison with established clinical prognostic models, calibration and clinical-utility analyses, and external validation.

## Conclusion

Baseline D-dimer, fibrinogen and APTT were associated with 90-day neurological functional status among AIS patients undergoing intravenous rt-PA thrombolysis after adjustment for the measured covariates included in the multivariable model. The exploratory four-marker model showed apparent discrimination within this single-centre cohort, but the findings do not establish that coagulation markers alone predict outcome or that they provide incremental clinical utility beyond established factors such as age and stroke severity. These results should therefore be considered hypothesis-generating until confirmed in larger prospective, externally validated cohorts, ideally with comparison against clinical-only models and with evaluation of calibration and clinical usefulness.

## Data Availability

The original contributions presented in the study are included in the article/Supplementary Material, further inquiries can be directed to the corresponding author.
